# Challenges towards the Sustainability and Enhancement of the Indian Sundarban Mangrove’s Blue Carbon Stock

**DOI:** 10.3390/life13081787

**Published:** 2023-08-21

**Authors:** Abhra Chanda, Anirban Akhand

**Affiliations:** 1 School of Oceanographic Studies, Jadavpur University, Kolkata 700032, West Bengal, India; 2 Department of Ocean Science, The Hong Kong University of Science and Technology, Clear Water Bay, Kowloon, Hong Kong SAR, China; 3 Coastal and Estuarine Environment Research Group, Port and Airport Research Institute, Nagase, Yokosuka 239-0826, Kanagawa, Japan

**Keywords:** salinization, freshwater scarcity, tropical cyclones, mangrove mortality, mangrove clearing, soil erosion, heavy-metal pollution, anthropogenic threats

## Abstract

The Sundarban is the world’s largest contiguous mangrove forest and stores around 26.62 Tg of blue carbon. The present study reviewed the factors causing a decline in its blue carbon content and poses a challenge in enhancing the carbon stock of this region. This review emphasized that recurrent tropical cyclones, soil erosion, freshwater scarcity, reduced sediment load into the delta, nutrient deficiency, salt-stress-induced changes in species composition, mangrove clearing, and anthropogenic pollution are the fundamental drivers which can potentially reduce the total blue carbon stock of this region. The southern end of the Ganges–Brahmaputra–Meghna Delta that shelters this forest has stopped its natural progradation due to inadequate sediment flow from the upper reaches. Growing population pressure from the north of the Sundarban Biosphere Reserve and severe erosion in the southern end accentuated by regional sea-level rise has left minimal options to enhance the blue carbon stock by extending the forest premises. This study collated the scholarly observations of the past decades from this region, indicating a carbon sequestration potential deterioration. By collecting the existing knowledge base, this review indicated the aspects that require immediate attention to stop this ecosystem’s draining of the valuable carbon sequestered and, at the same time, enhance the carbon stock, if possible. This review provided some key recommendations that can help sustain the blue carbon stock of the Indian Sundarban. This review stressed that characterizing the spatial variability of blue carbon with more sampling points, catering to the damaged trees after tropical cyclones, estuarine rejuvenation in the upper reaches, maintaining species diversity through afforestation programs, arresting coastal erosion through increasing sediment flow, and combating marine pollution have become urgent needs of the hour. The observations synthesized in this study can be helpful for academics, policy managers, and decision makers willing to uphold the sustainability of the blue carbon stock of this crucial ecosystem.

## 1. Introduction

It has been almost five decades since the human civilization on Earth recognized the ill effects of climate change and its manifestations. Efforts to curb anthropogenic greenhouse gas emissions are increasing manifold; however, much work is still needed to optimize an amicable solution to combat this evil [1]. The efficacy of nature-based solutions compared to engineered alternatives remains debatable [2]; yet, several scholars argue that the “Reducing Emissions from Deforestation and Forest Degradation (REDD)” framework has substantial potential to mitigate climate change [3]. Marine ecosystems, in this regard, have long received special attention due to their high carbon-sequestration potential compared to most terrestrial vegetated ecosystems [4,5,6]. The term “blue carbon,” which denotes the carbon stock sequestered by the coastal vegetated ecosystems worldwide, became quite relevant since its inception in 2009 [7,8]. The marine scientific community is looking for several options that can effectively come under the umbrella of blue carbon; however, at present, only mangroves, seagrasses, and salt marshes qualify as conventional blue carbon ecosystems [9]. Well before the anthropogenic carbon emissions and consequent climate change were realized, scientists looked for marine carbon pools in phytoplankton and some vegetated undersea ecosystems, like the seagrasses [10,11]. Academics and policy managers strongly believe that the consortia of blue carbon ecosystems can act as an effective tool to combat climate change [12]. Thousands of scholarly articles unequivocally pointed out that the high primary productivity of the coastal vegetated ecosystems can bury carbon for centuries to millennia in their sediments, which, if left undisturbed, acts as a long-term carbon sink [13,14,15]. If the extent of healthy blue carbon ecosystems can be successfully enhanced, it can trap more anthropogenically emitted CO_2_ [16,17]. At the same time, if the degradation of such ecosystems is allowed to take place, it inadvertently emits CO_2_ into the atmosphere [18]. Thus, the simplistic idea is to conserve blue carbon ecosystems (to minimize emissions from these ecosystems due to their degradation) and, if possible, to enhance their extent, capture more CO_2_ from the atmosphere, and combat climate change.

Mangrove ecosystems are perhaps the most carbon rich of conventional blue-carbon ecosystems and deserve special mention owing to their carbon-storing capacity [19]. Mangroves are a specialized group of floras that can withstand saline conditions and thrive in the coastal and estuarine peripheries, particularly in the tropical and subtropical regions [20]. These plants are primarily evergreen and can store substantial quantities of carbon in their aboveground and belowground biomass [21,22]. The anaerobic conditions in the mangrove sediments and the overall hydrodynamics of these ecosystems facilitate storing vast amounts of carbon in their soils [23]. The sediment substratum in the mangrove environment can effectively stabilize the highly recalcitrant organic carbon that accumulates bulk carbon content through the varying depths of the soil if left undisturbed [24]. The year-round litterfall also substantially enhances the soil’s organic carbon pool [25]. The mangroves usually exhibit high photosynthetic carbon assimilation potential, high net primary productivity, and low soil respiration rates, mainly because of the anaerobic conditions [26]. All these factors make the mangroves one of the most effective marine carbon sinks in the tropics. The mangrove ecosystems provide humankind with various ecosystem services [27]. In several tropical countries, the coastal fishery resource depends substantially on the mangroves and their nutrient-rich water column [28]. Mangrove-based shrimp, crab, and prawn farming are also quite popular in various parts of the world, providing a livelihood to millions [29]. Providing natural protection from extreme physical forcing events like tsunamis and cyclones is a fascinating ecosystem service mangroves provide coastal communities [30]. Mangroves also play a pivotal role in conserving sediments and preventing erosion in the coastal periphery [31]. Mangrove-based tourism is yet another sector that is blooming to the current date and serves as the only means of livelihood for millions [32]. Earlier studies estimated that the total economic value of the global mangroves could be as much as USD 181 billion [33]. Debates remain over the monetary evaluation of the ecosystem services of mangroves; however, the present-day scientific community unequivocally acknowledges its overall significance and the need to conserve and protect this crucial ecosystem worldwide.

Even though mangroves provide a myriad of ecosystem services, including climate-change-mitigation potential, these ecosystems currently face a severe threat worldwide. Globally, this unique floral community encompasses 70 species, of which 40% are experiencing threats of extinction [34]. Mangroves, by nature, are adept at keeping pace with the regional sea-level rise; however, anthropogenic interventions like damming the rivers and constructing barrages bar the required amount of sediment from reaching the estuarine reaches, which leads to their deterioration in many regions of the world [35]. Anthropogenic clearing of mangroves for agricultural purposes and aquaculture creation is another alarming aspect that has led to most of the mangrove decline, especially in the mangrove-rich Southeast Asia [36]. Several scholarly articles indicated that physical forcing events, like tropical cyclones, which increase in intensity and frequency with time, leave perilous signatures in mangrove floristic stands [37,38]. With the ongoing human development, the water quality of several estuaries has deteriorated, as these regions receive all the runoff and discharge from the upper urbanized reaches [39]. The mangrove-adjacent estuaries are also no exception in this regard. Pollutants like heavy metals, persistent organic pollutants, and microplastics severely deteriorate the mangrove sediments and even hamper the physiological functioning of these tidal halophytic floras [40,41,42,43]. Recent research suggests that many pathogens can also lead to mangrove mortality [44]. Mangrove conservation and management have become a top priority due to all these natural stressors and anthropogenic threats [45]. Restoring the ecosystem services these floras provided and enhances their blue carbon stock has emerged as a challenge for environmental scientists and practitioners.

In this regard, the present study focussed on the largest mangrove forest in the world, the Sundarban, which encompasses an area of 10,000 km^2^, situated at the southern end of the world’s largest delta, the Ganges–Brahmaputra–Meghna (GBM) Delta, shared by the neighboring countries, India and Bangladesh. Sundarban mangroves are globally significant due to their rich floral and faunal species diversity. This unique eco-region shelters nearly half the known mangrove species worldwide [46] and is the only mangrove tiger habitat in the world [47]. It also shelters some globally endangered species like *Panthera tigris tigris*, *Platanista gangetica*, *Orcaella brevirostris*, *Crocodylus porosus*, and *Batagur baska*. In the late seventeenth century, this deltaic mangrove forest used to have four times the spatial extent it now has. Since the beginning of the colonial era, this region has experienced indiscriminate deforestation, primarily to meet timber requirements and agricultural purposes and to expand human settlements [48]. Even after India gained Independence in 1947, this region experienced rapid human encroachment through deforestation [48]. It was only after 1970 that several forest conservation initiatives undertaken by the Government of India, like the Wildlife Protection Act (1972), Forest Conservation Act (1980), and Environment Protection Act (1986), were able to arrest the deforestation rate in the Indian Sundarban. After becoming an independent nation in 1971, Bangladesh also formulated several development programs to safeguard the biological integrity of Sundarban through poverty alleviation [49]. However, despite several initiatives implemented by the Governments of India and Bangladesh, the Sundarban mangrove area declined in the past three to four decades due to multiple factors. Besides being an abode to several floras and faunas, Sundarban shelters more than 8 million people (combining estimates of Indian and Bangladesh counterparts), and this region is one of the most densely populated regions of South Southeast Asia [50]. Most of this population is marginalized and impoverished and depends on forest-derived ecosystem services to meet their livelihoods [51]. Besides the increasing human pressure, factors like regional sea-level rise, scarcity of freshwater, erosion, coastal flooding, tropical cyclones, embankment failures, and coastal pollution pose severe threats to the social–ecological equilibrium of this unique ecosystem [52]. The anthropogenic pressure from the north (landward end) and the rapidly aggressing seawater from the south (seaward end) are incessantly squeezing the spatial extent of Sundarban from both ends, raising questions about its future sustainability.

Sundarban has received substantial attention from the scholarly communities of varied disciplines that studied the diverse aspects, attributes, and drivers that jeopardize this ecosystem’s overall well-being. The blue carbon repository of Sundarban is also not an exception in this regard. The existing literature on the carbon stock of the Sundarban mangroves primarily focused on estimating the carbon stock in the different mangrove compartments, characterizing the carbon biogeochemistry, and studying the exchange of carbon between the atmosphere and this mangrove ecosystem [53,54]. However, these literature seldom discussed the problems in managing the blue carbon stock of this region. Many articles comprehensively discussed the fate of Sundarban mangrove floral stands due to climate change and anthropogenic threats; however, similar endeavours are absent for its blue carbon stock. The present review of the literature indicates a dearth of any study that summarized and discussed all the potential challenges to sustaining and enhancing the blue carbon stock of Sundarban. The information remains scattered in many scholarly articles, and no known attempts to collate this information under one umbrella exist. This knowledge gap compelled the author to formulate the central research question, “What are the fundamental problems and challenges towards the sustainability and enhancement of the Indian Sundarban mangrove’s blue carbon stock?”. In alignment with this proposed research question, the primary objectives of this review were to (i) collate all the plausible drivers that pose a threat to the sustainability and enhancement of the Indian Sundarban mangrove’s blue carbon stock, and (ii) offer potential recommendations that can be instrumental in future policy actions.

### 1.1. Indian Sundarban Mangrove Ecosystem—A Brief Overview

**Figure 1:**
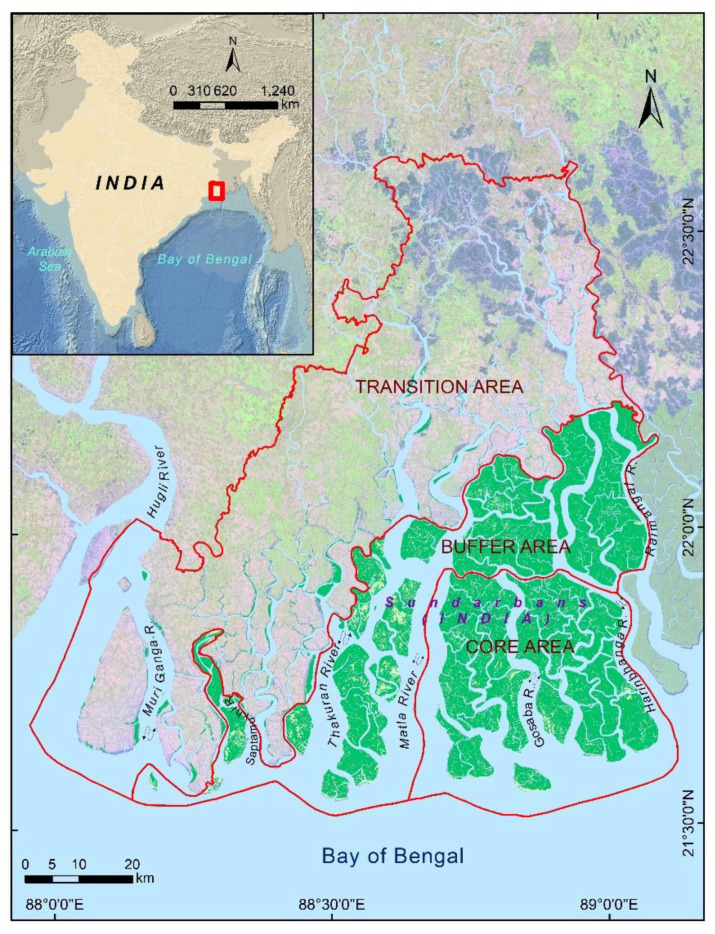
The core, buffer, and transition areas of the Indian Sundarban.

The present study dealt exclusively with the Indian part of Sundarban (Figure 1). An intricate mesh of estuaries forms the landscape where mangroves occupy discrete islands throughout this tail of the delta. To conserve the Royal Bengal Tigers, the Government of India declared the core area of the present Indian Sundarban as Sundarban National Park in 1984. UNESCO inscribed this region as a World Heritage Site in 1987. In 1989, the Government of India constituted the Sundarban Biosphere Reserve (SBR), and subsequently, in 2001, UNESCO recognized this endeavor under its Man and Biosphere (MAB) Programme. The SBR, at present, hosts around 4.4 million people. A substantial part of this population directly or indirectly depends on the Sundarban mangrove forest-derived products and services for their sustenance and primary livelihoods [55]. In 2019, the Sundarban was designated as a Ramsar site, an international treaty meant for the sustainable use and conservation of wetlands of global significance. This region enjoys a typical hot and humid tropical climate [56]. Precipitation in this part of the world mainly comes during the monsoon. June to September usually marks the monsoon season, followed by October to January as the post-monsoon season. The winter months of December and January experience the lowest temperature and humidity. February to May demarcates the pre-monsoon season, including the summer months of April and May, when the ambient temperature remains the highest. The estuarine complex of the Indian Sundarban comprises several large estuaries like Hooghly, Muriganga, Saptamukhi, Thakuran, Matla, Bidya, and Raimangal (from west to east). However, many estuaries lost their connection with the perennial flow of the Ganges and are on the verge of losing their estuarine character, especially during the dry seasons [57]. This region experiences semi-diurnal tides of varying amplitudes and strong tidal currents [58]. Oceanographers classify the Sundarban estuaries as meso–macro tidal ones. The mangrove forest of the Indian Sundarban comes under the purview of the West Bengal State Forest Department. They have segregated the entire biosphere reserve into three sectors: the core, the buffer, and the transition zones [59]. The transition zone is an abode for the 4.4 million residents, with very few fringe mangrove patches in some island peripheries. The core and buffer regions encompass the mangrove islands. The state forest department allows some activities, like fishing, crab collection, and tourism, in the buffer area; however, the core remains strictly restricted for civilians. A substantial part of the core overlaps with the Sundarban Tiger Reserve (STR), which came into operation in 1984, mainly to safeguard and conserve the Royal Bengal Tigers and their habitat. Besides the increasing number of residents in the SBR, millions of tourists visit Sundarban to cherish its landscape and wildlife [60].

#### Ecosystem Services Furnished by the Mangrove Stands of Sundarban

India continues to be an agricultural economy, and the Indian SBR is also no exception. Most people practice and rely on livelihoods derived from agriculture [61]. However, thousands of the residents of SBR depend on the mangrove forest for myriads of ecosystem services provided by these fascinating marine floras. Estuarine fishing is a decent livelihood for many in this region [62]. Several scholars argue that the mangrove-drained water from the Sundarban also plays an integral role in governing offshore fishing in the adjacent northern Bay of Bengal [63]. Fish, crabs, shrimps, and make up some of the direct provisioning services Sundarban furnishes to thousands [64,65]. Among the other non-timber forest products, honey deserves special mention, as Sundarban offers a sizable share of India’s total honey production [66]. Millions of tourists visit Sundarban each year to cherish its landscape and wildlife, as one can find cultural and aesthetic assets in this set-up. This region is particularly susceptible to tropical cyclones originating in the Bay of Bengal. The mangrove stands are a natural speed breaker to such storms and shelter both man and property from nature’s wrath [67]. Thus, the mangroves of this region are valuable to the locals as they protect themselves from strong winds and storm surges. Regulation of local climate, preventing coastal erosion, and combating regional sea-level rise, are some other services that the scholars have discussed in the past three decades. Besides the services mentioned above, Sundarban is a vital blue carbon sink that can significantly alleviate carbon emissions [68].

## 2. Materials and Methods

### 2.1. Review and Search Strategy

The Preferred Reporting Items for Systematic Reviews and Meta-Analyses (PRISMA) guidelines were followed to conduct the present review and subsequent meta-analysis of the collated data [69]. The author collated a set of key terms/index terms by searching the literature base with the keywords ‘Indian Sundarban’, ‘blue carbon’, and ‘mangrove carbon stock.’ Going through the initially downloaded 40 pieces of the literature, the author tried to develop an idea of the potential threats and challenges to the carbon stock of the Indian Sundarban and accordingly refined the additional set of index terms used for this review. Scanning through the initial set of papers, the author used the following index terms in addition to ‘Sundarban’ and ‘India’: ‘aboveground biomass’, ‘belowground biomass’, ‘soil organic carbon’, ‘mangrove species assemblage’, ‘ecosystem carbon stock’, ‘carbon sequestration potential’, ‘mangrove carbon’, ‘carbon biogeochemistry’, ‘tropical cyclones’, ‘storm surges’, ‘coastal erosion’, ‘coastal flooding’, ‘freshwater scarcity’, ‘salinization’, ‘mangrove mortality’, ‘sediment starvation’, ‘nutrient deprivation’, ‘anthropogenic disturbances’, ‘coastal pollution’, ‘aforestation’, and ‘mangrove restoration’. Google Scholar was used as the primary search engine, and the full papers were downloaded utilizing institutional access to ‘ScienceDirect’.

### 2.2. Paper Selection Criteria

**Figure 2:**
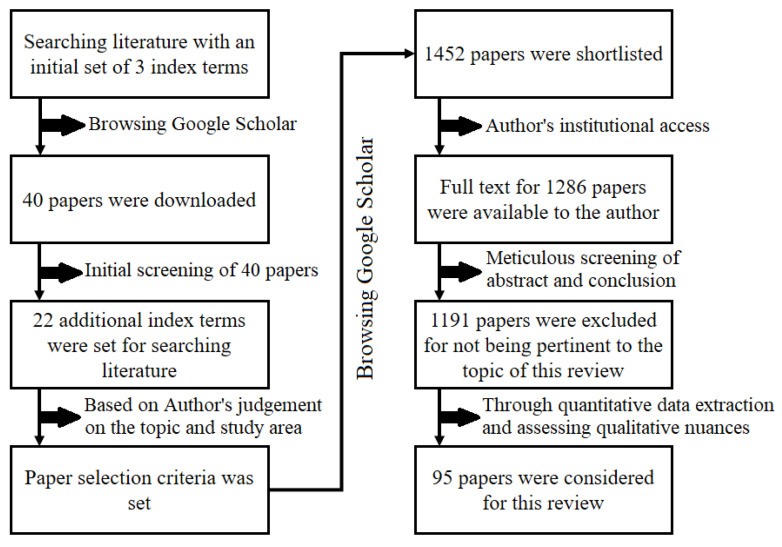
Flowchart of the methods adopted in the systematic review undertaken in this study.

Primary screening was carried out by reading the title and abstract of the papers. Based on this primary screening, the author shortlisted a set of potentially eligible research articles. Followed by shortlisting the papers, the full text of the articles was downloaded (Figure 2). The author critically reviewed whether or not the downloaded articles met the selection criteria. Only those papers were considered in this review that met all the below-mentioned inclusion criteria: (a) articles published in the English language, (b) articles that are original work (and not review papers), (c) articles for which the full text is available on the internet, and (d) articles published between the years 2000 and 2023. The term blue carbon came into existence in 2009; however, the author noticed several scholarly articles on the mangrove carbon stock of the Indian Sundarban since the onset of this century. Hence, the time frame of the paper search was deliberately kept between 2000 and 2023.

### 2.3. Data Extraction

The research question of this review did not only look for quantitative data but also tried to pinpoint the qualitative nuances concerning the threats to the sustainability of the blue carbon stock of the Indian Sundarban. However, whatever quantitative data have been collated in the paper, like mangrove biomass and soil carbon content, rate of sea-level rise, and mangrove area destroyed due to cyclones, have been considered only when these data were either mentioned in the text or tables. No image digitization technique was implemented in this study to digitize any data from any illustration or figure. The author judiciously extracted the qualitative aspects discussed in this review from the papers reviewed for this study.

## 3. Blue Carbon Stock—Knowledge Acquired So Far

**Figure 3:**
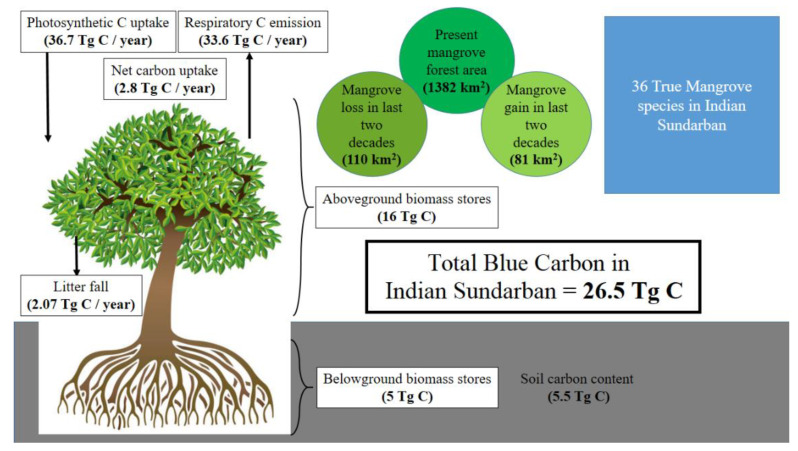
The blue carbon content of the Indian Sundarban and other associated statistics obtained from the secondary literature. The upward and downward arrows denote carbon efflux and influx from and to the biosphere, respectively.

Among the many attributes of the Indian Sundarban that are ecologically and environmentally significant and worth studying, carbon stock has perhaps received comparatively little attention. One of the reasons could be that the concept of blue carbon and the understanding of emission reduction through forest protection remains restricted within academia. The local communities value the mangroves for multiple purposes that directly relate to their lives, livelihoods, and safety; however, the global significance of these mangroves, from the perspective of being a solid repository of carbon, is yet to penetrate among the commons. Five significant studies reported measurements of carbon stock in the three fundamental compartments of a mangrove stand, the aboveground biomass (AGB), the belowground biomass (BGB), and the soil carbon pool (SCP). Most of these studies adopted the allometric approach for quantifying biomass carbon (Figure 3). Ray et al. [70] carried out a detailed carbon measurement covering all the compartments, and they observed 39.9 ± 14.1, 9.6 ± 3.4, and 17.4 ± 2.3 Mg C ha^–1^ in the AGB, BGB, and SCP, respectively. Banerjee et al. [71] and Mitra et al. [72] reported an SCP of 28.5 ± 2.0 and 20.4 ± 5.6 Mg C ha^–1^, respectively. Joshi and Ghose [73] also measured the AGB carbon content and SCP in the Indian Sundarban; however, they presented their observations as a range instead of mean ± standard deviation. The recent observations by Barik et al. [74] also conformed with the earlier findings; however, they did not report SCP per unit area. Besides these studies, four others [75,76,77,78] characterized the carbon stock in the Indian Sundarban; however, they focused on species-specific carbon stock instead of area-specific assessments. Lately, Akhand et al. [79] reviewed the blue carbon stock in the Indian coastal periphery and collated the observations from the Indian Sundarban in detail. Few of these studies derived a total aggregate carbon stock for the Indian Sundarban. Ray et al. [70] mentioned that the combined AGB and BGB (live) account for 21.13 Tg C. In contrast, the SCP holds around 5.49 Tg C. Barik et al. [74] also extrapolated their findings for the entire Indian Sundarban and reported a possible total carbon stock of 25.17 Tg C.

An absence of a robust estimate of the total blue carbon stock covering the entire Indian Sundarban is one of the first challenges to its sustainability. Estimating the blue carbon content of any marine region (with a higher degree of confidence) is of utmost importance. Efforts to conserve the carbon stock can only lead to meaningful and quantifiable outcomes if reliable knowledge exists on the amount of carbon that needs to be protected. Though several studies have drawn a holistic estimate of the mangrove blue carbon stock in the Indian Sundarban, uncertainties prevail for multiple reasons. Almost all the studies reported above have concentrated on the buffer region of the mangrove forest mainly because of the ease of access. Carbon stock estimates from the forest’s core area are hardly available. Entering the core area that overlaps with the STR requires permission from the state forest department and has intrinsic risks of conducting research. The absence of carbon stock data from the core introduces substantial uncertainty in deriving the total aggregate carbon stock magnitude. The measurement protocol of SCP varies across different schools of researchers. Some studies considered the top 30 cm, whereas others focused on one-meter topsoil. Thus, deriving an area-specific unit of SCP remains a challenge. Given the spatial extent of the Indian Sundarban, much more high-resolution ground sampling at a time is necessary to quantify this region’s total blue carbon stock with a higher degree of confidence. Though several studies pointed out that the genus *Avicennia* primarily dominates the Indian Sundarban mangrove stands, it is still a heterogenous cover, which requires more sampling to account for the small-scale spatial variabilities in the carbon stock across the breadth of this mangrove forest. The State Forest Department officials (who have access to the entire Sundarban forest, including the core area) should join hands with academia to derive a reliable estimate of total blue carbon for this region.

## 4. Strong Physical Forcing Events—Tropical Cyclones

**Figure 4:**
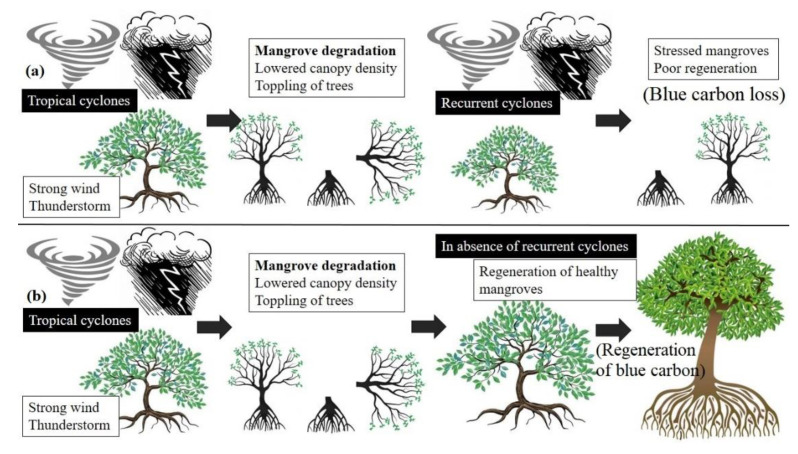
The effect of (**a**) recurrent and (**b**) isolated tropical cyclones on the mangrove floral stands of the Indian Sundarban.

Tropical cyclones are common in the Bay of Bengal. Meteorological records show several storms formed in this region in the past centuries. Many made landfall in the West Bengal (India) and Bangladesh coastline guarded by the dense mangrove stands of Sundarban [80]. The severity of the mangrove destruction usually depends on the intensity of the tropical cyclones, especially the landfall speed. Though the sea-facing mangroves are accustomed to high wind speed, extreme physical forcing events like cyclones often topple down several trees and break the large branches in the aboveground shoot and trunk system [81]. Tropical cyclones are not new to the mangroves of Sundarban, and these unique halophytic species have shown a substantial potential to regenerate on their own after such events [82]. However, several scholars have argued that due to the rise in global temperature, the frequency and intensity of these atmospheric disturbances are likely to increase in the Bay of Bengal [83,84]. Recent studies indicated that the surface layers of the Bay of Bengal are anomalously warming compared to the earlier days up to a 600 m water depth, favoring frequent cyclone genesis in this basin [85]. Mangrove specialists argue that increasing the frequency and intensity of these disturbances would incur more significant damage to the mangroves and offer less time to recuperate from such atmospheric shocks (Figure 4).

In November 1970, a deadly supercyclone, “Bhola,” landed in the Bangladesh Sundarban region, leading to more than 300,000 casualties and severe loss of properties. Several scholarly articles discussed the loss of lives and livelihoods primarily due to this deadly cyclone [86,87]; however, comments on mangrove destruction are scarce. Recently, the mangrove stands have started getting the attention of scholarly communities after such atmospheric shocks. Some cyclones directly hit the Bangladesh Sundarban, whereas some pass through the Indian counterpart. Despite the exact location of the landfall, these cyclones usually leave disastrous signatures on the mangroves of both nations. Thus, this review discussed all the significant cyclonic impacts observed by the scholars of India and Bangladesh. Mandal and Hosaka [80] collated that cyclone “Sidr” in November 2007 impacted almost 1291 km^2^ (24% of the combined forest area of Indian and Bangladesh Sundarban) of the Sundarban mangrove forest. The comparatively taller genera, like *Sonneratia* sp., bore the wrath of the strong winds [88]. The following year, cyclone “Rashmi” landed on the Sundarban; however, the impact was less prominent due to a lesser wind speed than Sidr [89]. Followed by Rashmi, 2009 witnessed another devastating cyclone, “Aila”, that led to considerable loss of mangroves and significantly hampered the lives and livelihoods of thousands in the following years. The Indian Sundarban lost around 21.6 km^2^ of the forested area [90]. In 2015 and 2016, cyclones “Komen” and “Roanu” severely affected 137 km^2^ and 152 km^2^ of Sundarban mangrove forest, respectively [80]. Cyclone “Bulbul” and very severe supercyclone “Amphan” in 2019 and 2020, respectively, substantially reduced the mangrove canopy cover of the Indian Sundarban [91]. Mishra et al. [92] documented that the dense mangrove cover of Sundarban shrank from 77% to only 34% after the Amphan cyclone. Recently, cyclone “Yaas” in 2021 led to widespread inundation in the coastal state of West Bengal; however, the impacts on mangroves are yet to be studied [93]. This series of observations (Table 1) indicates that the Sundarban experiences an intense atmospheric disturbance almost yearly. The strong wind becomes detrimental for the mature trees, and the saline water intrusion and subsequent inundation impart salt stress on the oligohaline mangrove species. The salt spray induced by such physical forcing events leads to several mangrove trees’ mortality, especially those on the island peripheries. Mangrove destruction due to such natural phenomena leads to a significant loss of blue carbon from this forest. The aboveground biomass getting ravaged eventually leads to CO_2_ emission through the decay and mineralization of the dead mangrove remains. Thus, tropical cyclones’ increased frequency and intensity can be a challenge to sustaining the blue carbon stock, especially in the live biomass of the mangrove floral stands. The relentless efforts of the West Bengal State Forest Department officials overseeing the Indian Sundarban deserve all the praise. However, they should strengthen their damage recovery team to cater to all the mangrove trees immediately after cyclonic hazards. Adequate pruning of damaged trees, covering exposed roots with soil, and clearing broken and malformed branches can help many mangrove trees recover quickly from cyclone-induced damage and regenerate on their own.

## 5. Salinization of Estuaries and Mangrove Sediments

**Figure 5:**
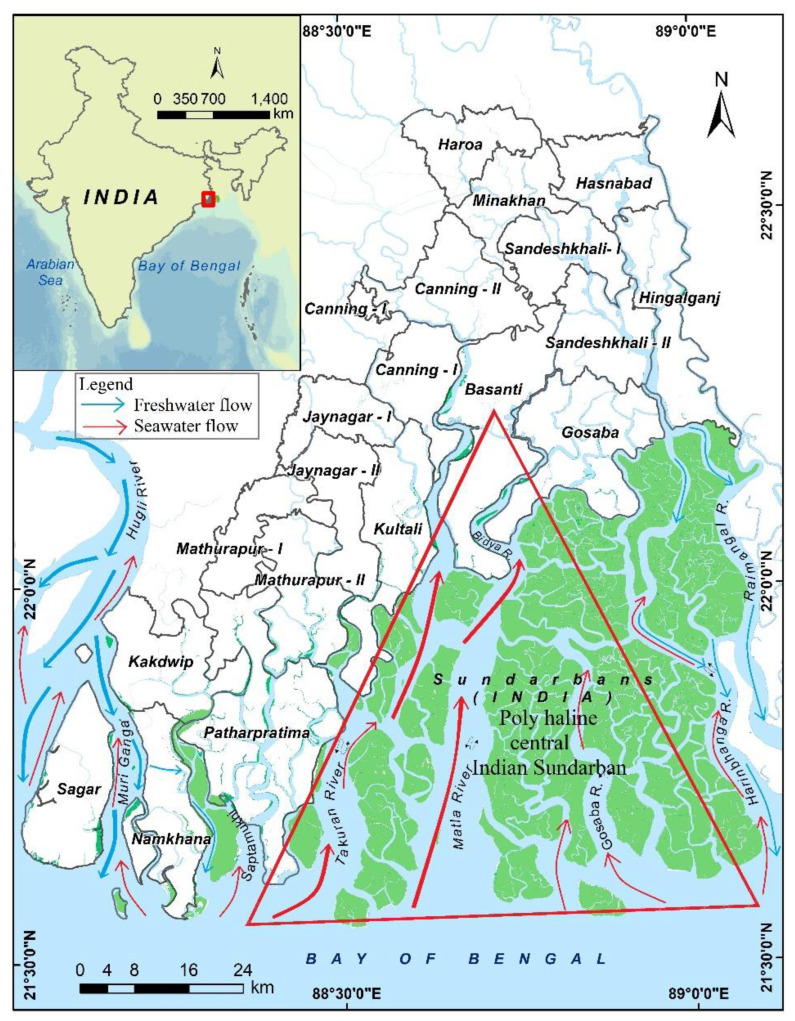
Location and map of the central part of the Indian Sundarban (red triangle) associated with the dominance of seawater and scarcity of riverine freshwater that is primarily responsible for the changes in mangrove species assemblage.

Mangrove floral species are salt tolerant and require saline water for their growth and flourishment; however, the degree of salt tolerance varies across species diversity, and at times, excess salinity can be detrimental for mangroves, as well [94]. Several studies have indicated that Sundarban estuaries and the sediment column currently suffer from salinization [95,96]. Mitra et al. [97] reviewed the salinity data of the last six to seven decades in and around the Indian Sundarban estuaries and constructed temporal trends in salinity changes. They observed that the Hooghly Estuary flowing through the western boundary and the Raimangal Estuary flowing through the east are the only perennial freshwater sources into the Sundarban mangroves. The estuaries in the central portion of this ecosystem, which confines most of the mangrove islands of Sundarban, receive a minimal freshwater discharge from the upper reaches (Figure 5). The seawater encroaching during the flood tide dominates these estuaries all year round. Mitra et al. [97] computed that in the last three decades, several regions in the central part of the Indian Sundarban experienced an increase in salinity of 0.12 to 0.88 per year. The higher residence time of seawater within the estuarine reaches, as observed by Akhand et al. [98], might be responsible for these elevated salinity levels. Mitra et al. [99] attributed the heavy siltation in the upper reaches of the central estuaries to the increase in salinity of this region. The heavy siltation in the upper reaches blocks adequate freshwater supply to the estuaries of the Indian Sundarban, leading to a gradual increment in salinity over time. Chatterjee et al. [57] also indicated that human encroachment, heavy siltation, and disconnected waterways in the upper reaches of the central-flowing estuaries of Sundarban have led to the acute scarcity of freshwater in these estuaries. Chowdhury et al. [100] indicated that regional sea-level rise could be another crucial factor leading to the sharp rise in salinity in the Indian Sundarban. Mondal et al. [101] pointed out that the regional sea-level rise rate in Sundarban, which used to be 1.7 mm yr^−1^ in the nineteenth century, has escalated to 3 mm yr^−1^ by the end of the twentieth century.

Alterations in the salinity regime and excess salinity in the estuarine water mass irrevocably affect the mangrove-adjacent sediments and alter the soil salinity, affecting mangrove growth dynamics [102]. Ray et al. [70] observed that salinity contributed 26.2% in governing the biomass and carbon stock in the Indian Sundarban. Chowdhury et al. [103] sampled several intact and degraded mangrove sites of the Indian Sundarban. They inferred that excessive salt accumulation in the sediment column led to high osmotic potential, resulting in low carbon flow in the sediments and affecting mangrove growth. The enhanced salinity levels also hampered the soil’s enzyme activities [104]. The disturbed forest regions exhibited exceptionally high salinity, which led the scholars to conclude that the increase in salinity plays a crucial role in degrading the mangrove forest cover in the Indian Sundarban. Banerjee et al. [96] indicated that salinity was pivotal in regulating the biomass of *Heritera fomes* in Sundarban. Regions with high salinity showed a lower biomass of this species and vice versa. Banerjee et al. [75] also observed a significant negative correlation between salinity and the biomass of an oligohaline species, *Sonneratia apetala*, across the spatial extent of the Indian Sundarban. Dasgupta et al. [105] observed that several oligohaline mangrove floras showed lesser protein content in leaves with increasing salinity. Analyzing the degree of presence of reactive oxygen species, Dasgupta et al. [105] inferred that species like *Heritiera fomes*, *Xylocarpus mekongensis*, *Xylocarpus granatum*, and *Aegialitis rotundifolia* have a lesser tolerance towards increased salinity, and hence cannot survive under extreme salt stress. Nandy et al. [106] explored that several oligohaline species of the Indian Sundarban exhibited significantly lower photosynthetic carbon accumulation potential under salt-stressed conditions. All these observations indicate that the altered freshwater discharge from the upper reaches, anthropogenic river encroachment, heavy siltation, and regional sea-level rise has led to an enhanced salinity level in various regions of this mangrove forest. If this trend continues in the business-as-usual scenario or gets elevated under the climate change scenarios, it would significantly reduce the blue carbon strength of this region [107], principally by affecting the growth dynamics and photosynthetic carbon assimilation potential of the oligohaline species. Under such circumstances, channeling freshwater from the upper reaches of Hooghly to the central part of the Indian Sundarban, even through an underground canal network (if possible), has become an utmost necessity.

## 6. Changes in Mangrove Species Assemblage

The Sundarban furnishes a complicated network of estuaries flowing through a vast area that allows varying salinity zones, which was fundamental in facilitating the species diversity in this region. Sundarban exhibits a rich mangrove floral species biodiversity, and these species require varying degrees of salinity to thrive and perform at their fullest. Usually, the inner estuarine regions close to abundant freshwater resources furnish a suitable habitat for species that thrive well in low-saline conditions (oligohaline mangroves) [108]. The middle reaches that exhibit moderate salinity allow the mesohaline mangroves to thrive and flourish. In contrast, the highly saline outer reaches enable the polyhaline mangroves to grow well, which can withstand high salinity [109]. Barik et al. [110] explicitly discussed the salinity-based variability in species assemblage in the Indian Sundarban. They and many other studies indicated that genera like *Heritiera*, *Nypa*, *Sonneratia*, and *Lumnitzera* thrive under the oligohaline conditions of Sundarban. In contrast, *Exoecaria*, *Aegiceras*, *Kandelia*, *Aegialitis*, *Xylocarpus*, and *Bruguiera* prefer to grow in mesohaline regions. Polyhaline regions shelter those genera that withstand high salinity, like *Avicennia*, *Rhizophora*, *Ceriops*, *Phoenix*, etc. Some species, like *Avicennia marina*, *Avicennia officinalis*, and *Excoecaria agallocha*, dominate the floral stands of Sundarban, irrespective of the different salinity regimes.

**Figure 6:**
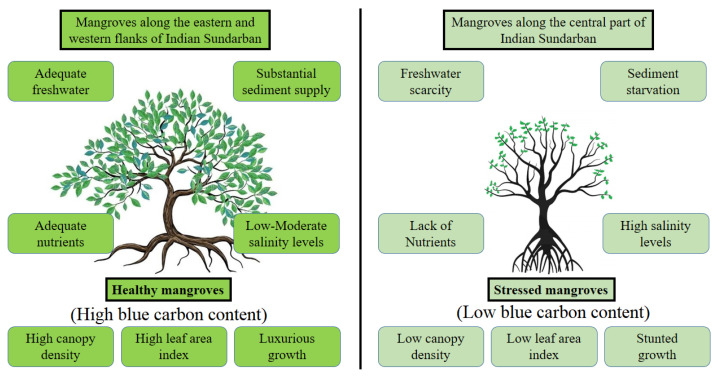
The spatial difference in salinity and its effect on the mangrove stands of the Indian Sundarban.

Several studies showed that the Sundarban is experiencing a significant shift in species assemblage primarily due to the changes in the salinity regime (Figure 6). Anecdotal information indicates that the term Sundarban originated from the name of the species *Sundari* (*Heritiera fomes*), meaning beautiful, and *bon* means forest. This species used to dominate this region; however, patches of natural stands of *Heritiera fomes* are scarcely available in the Indian Sundarban. Banerjee et al. [96] showed that the *Heritiera fomes* rapidly disappear in regions where salinity remains elevated throughout the year. Chowdhury et al. [103] observed that less salt-tolerant species, like *Aegialitis rotundifolia*, *Aegiceras corniculatum*, *Heritera fomes*, and *Xylocarpus mekongensis*, are diminishing throughout the Indian Sundarban at the expense of high salt-tolerant species like *Excoecaria agallocha*, *Avicennia marina*, *A. alba*, and *A. officinalis*. Chowdhury et al. [103] further observed that climax species like *Pheonix paludosa* decreased significantly in the Indian Sundarban and were absent in sediments having salinity > 6.5 ppt. Overall, species richness and evenness are declining in the Indian Sundarban, as only the genera *Avicennia* and *Excoecaria* are gradually replacing a myriad of less salt-tolerant mangrove species in this region, as these genera can acclimatize under a varying range of salinity [111]. Mukhopadhyay et al. [112] projected the future species assemblage in the Indian Sundarban for the year 2050. They inferred that if the salinity keeps increasing in this way, fewer salt-tolerant species will replace a much higher number of oligohaline species all through the Indian Sundarban, compromising its overall species biodiversity. Nandy and Ghose [113], while characterizing the photosynthesis efficiency of the dominant mangroves of the Indian Sundarban, observed that *Heritiera fomes* had the highest photosynthetic CO_2_ assimilation potential (13.21 ± 3.57 µmol m^−2^ s^−1^), which is gradually vanishing throughout the Sundarban due to the ongoing salinization. They also observed that species like *Excoecaria agallocha*, which is replacing this oligohaline species, had much less photosynthetic CO_2_ assimilation potential (varying between 5.57 ± 3.12 and 8.47 ± 7.00 µmol m^−2^ s^−1^). Mukhopadhyay et al. [114] implied cellular automata and Markov chain models to predict the mangrove species assemblage scenario in the Bangladesh Sundarban, which is also undergoing steady salinization of the estuaries. They observed that freshwater-loving genera like *Ceriops*, *Heritiera*, and *Xylocarpus* would steadily disappear with a concomitant increase in *Excoecaria agallocha*. Chanda et al. [115] worked on the same issue and computed that the Bangladesh Sundarban would lose 22.42 Tg C due to the depletion of oligohaline mangroves, which could cost approximately 58.28 Tg CO_2_ emission by the year 2105. Such an estimation for the Indian counterpart of the Sundarban does not exist at present; however, allied research indicates that the future will witness a substantial loss of blue carbon from the Indian Sundarban, as well, due to this change in species composition. These observations warrant maintaining the species diversity through restoration programs with particular emphasis on oligohaline species.

## 7. Erosion, Sediment Starvation, and Nutrient Deprivation

**Figure 7:**
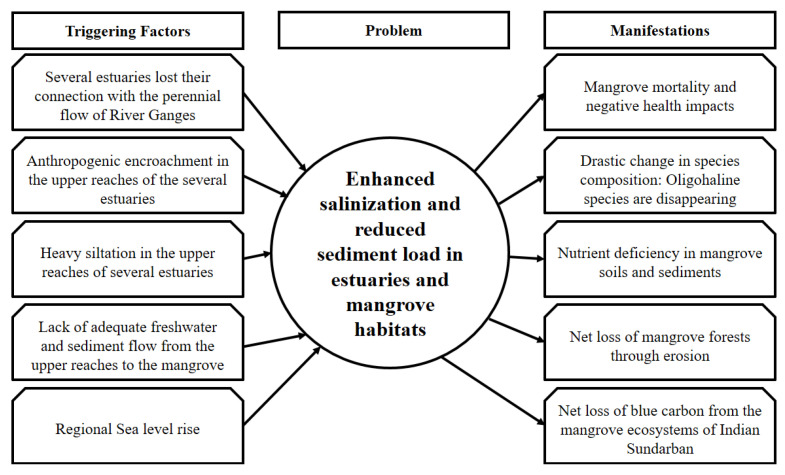
The fundamental drivers of enhanced salinization and reduced sediment flow into the Indian Sundarban and their impacts on this mangrove ecosystem.

Erosion and accretion are natural processes that take place incessantly in any deltaic region. However, sea-level rise and land subsidence, coupled with various anthropogenic activities, have disturbed the geomorphic balance of several large deltas. The GBM delta is no exception in this regard [116]. A net surplus of accretion over erosion is essential for any delta to prograde and facilitate an enhancement in coastal vegetation cover; however, several studies have unequivocally indicated that the Indian Sundarban is undergoing a steady decrease in the area (Table 2). Giri et al. [117], while characterizing the collective mangrove dynamics of India and Bangladesh, observed minor changes in forest cover between 1973 and 1990; however, between 1990 and 2000, they noted a significant decrease in forest cover. They pointed out that the top-dying disease and anthropogenic forest clearing are the prominent threats to the mangroves of this region. Akhand et al. [118] assessed the loss in the mangrove forest area between 1975 and 2013 in the Indian Sundarban. They observed that 60% of the net loss was due to erosion, followed by 23% due to mangrove thinning in many areas throughout the Sundarban. Anthropogenic conversion of mangrove areas to aquaculture, agriculture, and settlements are other potential factors that led to this system’s loss of blue carbon. In this context, Bhargava et al. [119] pointed out that a massive reduction in sediment supply to the deltaic reaches of Sundarban is a fundamental reason behind the loss of mangrove islands. Mangrove vegetation can consolidate deltaic sediments and help in soil conservation by slowing down water currents [120]; however, Samanta and Hazra [121] observed that the southern sea-facing islands that have mangrove vegetation and no anthropogenic vegetation perturbance have suffered the most intense soil erosion in the Indian Sundarban. This observation further suggests that under an utter lack of sediment supply, the mangrove roots that are otherwise responsible for soil compaction cannot withstand erosion due to wave-induced disturbances. In this regard, Thakur et al. [122] indicated that regional sea-level rise had introduced sandy topsoil layers in many regions where mangroves cannot cope to thrive. Mangrove mortality has also led to the conversion from vegetated regions to bare mudflats. They further commented that sea-level-rise-induced salinization is also responsible for mangrove thinning in several regions of Sundarban. Samanta et al. [91] added that phenomena like increasing ambient temperature and reduced rainfall in pre-monsoon and post-monsoon seasons are also drivers causing mangrove degradation in the Indian Sundarban. Aside from providing the physical substrate base, sediments play a crucial role in regulating the organic carbon, nutrient content, and enzymatic activities which govern mangrove growth and productivity [123,124]. Cannicci et al. [125] pointed out that allochthonous inputs from upper riverine reaches are pivotal in regulating the nutrient dynamics of mangrove soils. Chowdhury et al. [103] observed that a lack of freshwater and sediment from riverine reaches has led to nutrient starvation in the Indian Sundarban, which correlated well with poor mangrove health. They further indicated that the mangrove sediments of Sundarban lacking nutrient supply from upper reaches are undergoing nutrient washing due to tidal activities, which also wash away the autochthonous nutrients from litter degradation (Figure 7). Thus, sediment starvation is accelerating erosion and degrading the nutrient levels in the mangrove sediments, ultimately leading to the loss of blue carbon from this crucial ecosystem.

## 8. Anthropogenic Disturbances and Coastal Pollution

The upper reaches of the Sundarban estuarine systems and their catchment area encompass some of this country’s highly urbanized cities, metropolises, and industrial belts. The increasing population in the SBR has been enhancing the stress on the natural landscape of this area for decades [126]. The conversion of mangrove lands to agricultural plots and, recently, aquaculture farms have been the fundamental reason for mangrove degradation in the Indian Sundarban, especially in the peripheral regions between the transition and buffer zones of the forest [127,128]. The mangrove forest used to extend to the present Kolkata metropolis in the late eighteenth century, which began experiencing forest clearing in 1781 mainly to facilitate agriculture [129]. The present state forest administration has restricted human encroachment south of the buffer periphery; however, the fringe mangroves in the human-inhabited transition zone witnessed indiscriminate destruction recently. In the present scenario, where the delta has stopped prograding due to the lack of sediments from upper reaches, human encroachment from the south leaves no space for natural mangrove regeneration, which is the prime concern for this unique region in the longer run. Under such circumstances, mangrove conservation should remain at the forefront of policy making to ensure the existing blue carbon stock; however, enhancing the blue carbon stock remains challenging for policy managers.

**Figure 8:**
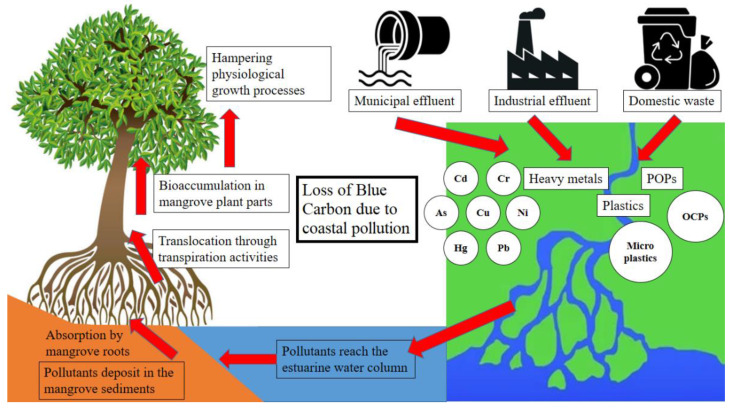
Schematic representation of the anthropogenic polluting activities in the upper reaches that eventually affect the Indian Sundarban mangroves.

Besides forest clearing to meet various anthropogenic demands and requirements, polluting activities also heavily affect the mangroves and their habitats (Figure 8). Untreated effluents from industrial belts have polluted this region heavily with heavy metals like arsenic, cadmium, cobalt, chromium, copper, iron, manganese, mercury, nickel, lead, and zinc [130]. Mangrove plants and sediments act as natural filters and tend to trap these metals from the adjacent estuarine water mass. Several scholars eyed this phenomenon as a means of phytoremediation [131]. Many studies have pointed out that excessive accumulation of heavy metals in different strata of this unique ecosystem can pose severe threats to the biodiversity of this region [132]. Characterizing the human health risk from such pollution has received the most attention [133]. However, few studies indicated that heavy-metal accumulation in mangrove plant parts could significantly alter their physiological functioning, directly impacting their health and growth rate [134]. Accumulating heavy metals in mangrove plants beyond a threshold can reduce their photosynthetic efficiency, which might gradually cause metal-sensitive species to disappear at the cost of those species resistant to metal accumulation [135]. Some Indian Sundarban mangrove plants showed that heavy metals like cadmium and mercury accumulated in hypodermal and stelar portions of the roots and stems, which led to the deformation of the xylem and phloem [136]. Thus, all these observations show that, in the longer run, heavy-metal pollution can be responsible for the decline in the blue carbon content of this ecosystem. Besides heavy metals, the Indian Sundarban mangroves exhibit significant polluting signatures of persistent organic pollutants; however, most studies focused on the sediment and biotic communities within the mangrove ecosystems [137]. Research focusing exclusively on the ill impacts of these pollutants on the photosynthetic carbon assimilation potential of the mangrove stands should expedite the development of a holistic understanding of this issue. Direct evidence of heavy metal, persistent organic pollutant, and plastic pollution impacting the blue carbon stock of the Indian Sundarban are not available in the scientific literature base. However, the pollutants mentioned above in this mangrove forest’s biotic and abiotic elements jeopardize the ecosystem’s overall ecological integrity. The lesser the ecological value of an ecosystem, the more difficult it is to conserve it. Hence, pollution abatement is crucial to uphold the pristineness of the mangrove floral stands and, thus, ensure their blue carbon stock.

## 9. Fate of Blue Carbon Stock under the Changing Climate and Environment

Owing to the ongoing climate change and anthropogenic emission of CO_2_, the global ambient temperature and atmospheric CO_2_ concentrations are expected to rise, followed by shifts in rainfall patterns, by the end of the twenty-first century [138,139,140]. Endeavors of modeling the impacts of such changes on the future of the carbon stock of Sundarban were scarce in the existing scientific literature base. Ray et al. [141] modeled the effect of CO_2_ enrichment in the atmosphere on the carbon stock of the Indian Sundarban and observed that the carbon in the live biomass and soils would increase by 1.10 and 1.57 times, respectively, if the atmospheric CO_2_ concentration reaches 580 ppmv. Ray and Jana [142] pointed out that the carbon sequestered by Sundarban mangroves is equivalent to the CO_2_ released by the nearest powerplant and almost 0.64% of India’s total coal-based CO_2_ emission. Chatting et al. [143] recently indicated that under both business-as-usual and high-emission scenarios, the global mangrove carbon stock could increase by 7 to 10% by the end of the twenty-first century. This observation indicates that the existing blue carbon stock of the Indian Sundarban assessed at 26.5 Tg C might enhance to 28.4 to 29.2 Tg C by 2100, provided deforestation remains arrested for the following years of this century. According to the Paris Agreement (2015), one of India’s pledges as a part of their nationally determined contribution (NDC) was to create an additional carbon sink of 680 to 820 Tg C by 2030. Thus, conserving the present mangrove extent of Sundarban can help fulfill ≈ 0.4% of India’s NDC. However, increasing global temperature would also increase the water surface temperature in the mangrove-adjacent estuaries and the soil temperature in the mangrove forests, affecting the biogeochemical fluxes. Carbon-biogeochemistry-centric studies in the Indian Sundarban estuaries indicated a significant positive correlation between the air–water CO_2_ efflux and estuarine water surface temperature [144]. Chanda et al. [145] observed an exponential relationship between mangrove soil temperature and soil CO_2_ respiration in the Indian Sundarban and reported that soil CO_2_ emissions could be four times higher if the soil temperature increases by 10 °C. These observations indicate that increased global temperature (besides enhancing the primary productivity of the Indian Sundarban mangroves) would also likely enhance CO_2_ respiration from the pedosphere and hydrosphere adjoining the mangrove stands.

The changing freshwater–seawater equilibrium in the estuaries of Sundarban, due to the lack of freshwater from the upper reaches, can significantly alter the lateral fluxes of dissolved organic carbon (DOC), dissolved inorganic carbon (DIC), and particulate organic carbon (POC). Ray et al. [146] indicated that mangroves of Sundarban export around 7.3 Tg C per year to the adjacent coasts of the Bay of Bengal. As the concept of blue carbon is evolving, many scholars pointed out that the lateral export of carbon to the offshore regions could be a significant carbon sink compared to carbon burial within the blue carbon ecosystems [147,148]. However, if the freshwater flow into the Indian Sundarban continues to deplete, especially from the eastern border, which drains through the entire core area of mangrove forests, the export of carbon from the mangrove forest to the adjacent Bay of Bengal would significantly decrease. As freshwater from the upper reaches usually exhibits low pH that facilitates the degassing of CO_2_ from the estuarine regions of Sundarban [149], the reduced freshwater supply enhances the dominance of seawater from the Bay of Bengal, which has a high carbonate buffering capacity (as evidenced from total alkalinity/DIC ratio and Revelle factor) in the Sundarban mangrove estuaries [150]. Owing to this high buffering capacity, the outer estuarine regions of the Indian Sundarban act as a sink for CO_2_, unlike most mangrove-adjacent estuaries of the world [150], and the riverbed sediment of Sundarbans stores more mangrove-derived blue carbon than that of riverine freshwater-dominated mangrove estuary [151]. Some scholars indicated that the frequency and intensity of tropical cyclones originating in the Bay of Bengal that often makes landfall on the Sundarban might continue to increase [152]. Tropical-cyclone-induced mangrove mortality can significantly reduce the blue carbon stock of the Indian Sundarban as the strong wind mostly topples down the mature mangrove trees. Sahu and Kathiresan [85] indicated that the older the mangrove trees, the higher the net canopy photosynthesis rate, implying higher carbon sequestration and blue carbon stock. Thus, various manifestations of climate change can increase and decrease blue carbon stock in the Indian Sundarban in multiple ways. Such observations warrant more holistic studies considering all the plausible drivers to identify the future challenges towards sustaining the blue carbon stock of this region.

## 10. Social–Ecological Challenges and Mangrove Restoration

Many people of the Indian Sundarban depend on the ecosystem services provided by the mangroves of this region. Estuarine fishing, crab collection, and honey collection are the principal means of livelihood for many people in this region. Besides income generation, the Sundarban mangroves help the residents combat the wrath of tropical cyclones. Several studies indicated that the lesser the local population’s forest dependency, the better the conservation and management of the forest. However, using forest-derived products without harming the mangroves should not affect this region’s biomass and blue carbon content. The lack of law enforcement often leads to illegal timber extraction [153], which directly impacts the blue carbon repository, and is one of the prime challenges to its sustenance. The engagement of local communities in conserving and safeguarding the mangroves is essential to maintain the carbon stock and the ecosystem services of this region for future generations. Banerjee et al. [154] pointed out that the carbon repository of the Indian Sundarban, if properly utilized, can provide significant monetary benefits to the local communities by selling carbon credits. However, initiatives to utilize the Clean Development Mechanism tool of the United Nations through the generation and subsequent trading of carbon credits by mangrove conservation are absent in the Indian Sundarban to date. Implementing such mechanisms can provide alternative means of livelihood to many and such endeavors would develop a sense of ownership among the locals and ignite a proactive mangrove conservation attitude among the local communities.

Despite several challenges that threaten the blue carbon stock of the Indian Sundarban, it witnessed few notable restoration endeavors in the recent past. Dey and Kar [155] mentioned that a lack of scientific approach, documentation of good practices, poor involvement of local communities, and improper long-term monitoring mechanisms lead to most restoration endeavors as fragmented and sporadic without any long-term benefits. However, Vyas and Sengupta [156] mentioned that the West Bengal Forest Department strives to build healthy relationships with local communities by forming Joint Forest Management Committees, especially in villages peripheral to the mangrove forest comprising Ecodevelopment Committees and Forest Protection Committees. The COVID-19 pandemic-induced lockdown led to a severe economic fallout among the local communities of the Indian Sundarban, and the concurrent tropical cyclone, Amphan (May 2020) impacted 43% of the Indian Sundarban mangroves [92]. Following this cyclonic storm, the Government of West Bengal called for a large-scale mangrove restoration plan covering 217 sites (encompassing 4219 ha) and planted 123.77 million mangrove saplings by March 2022 [154]. This restoration program effectively utilized a national government welfare scheme (Mahatma Gandhi National Rural Employment Guarantee Scheme: MGNREGS) to pay wages to local people involved in this program. Such an initiative was a respite for many local people to overcome the financial fallout. The restoration program successfully maintained the species diversity, performed nursery operations in an eco-friendly manner, and executed the entire operation borne out of a political and administrative will. However, Banerjee et al. [154] mentioned that lack of hydrodynamic monitoring before selecting the sites or species, not involving academia from the conceptualization stage, participation of women through a top-down approach, and uncertainties of funds for the long-term monitoring of planted saplings might undermine the success of such a large-scale endeavor. Thus, restoration programs in the Indian Sundarban as a nature-based solution to sustain the blue carbon stock are partially successful, with ample scope for improvements to eliminate chances of failure in the longer run.

## 11. Conclusions

Collating all the observations reviewed in this study, it is clear that the mangrove floral stands of the Indian Sundarban are under severe threat due to various natural and anthropogenic factors that substantially compromise the blue carbon stock of this region. The increasing frequencies and intensities of recurrent tropical cyclones make it difficult for the natural mangrove stands to regenerate. Freshwater scarcity, especially in the central mangrove-dense regions of the Indian Sundarban, has been enhancing the soil salinity beyond tolerable limits for many mangrove species that can store substantial carbon in their aboveground and belowground compartments. Lost connections of most estuaries with the perennial flow of the Ganges vis-à-vis the anthropogenic encroachment and heavy siltation in the upper estuarine stretches gradually render this region hypersaline, which is detrimental for several mangrove species. Most oligohaline mangrove species that cannot withstand such elevated salinity store higher carbon than the polyhaline salt-tolerant mangroves. Unfortunately, owing to the increased salinity levels, the polyhaline mangroves dominate at the expense of oligohaline ones, eventually declining this region’s net blue carbon stock. A lack of sediment flow from the upper reaches has restricted the progradation of this delta, and the present mangrove islands are facing a constant challenge to combat the ongoing processes like erosion and net land loss, which remains accentuated by regional sea-level rise. Besides reducing the salinity stress, the riverine freshwater brings in substantial quantities of allochthonous nutrients, which the gushing sea water can never compensate. Thus, the absence of adequate freshwater is the fundamental cause behind the degradation of mangrove soil quality. Several species are unable to survive under such nutrient-poor conditions. The mangrove health and growth rate directly proportional to the system’s total blue carbon content significantly deteriorates due to the lack of freshwater, sediments, and nutrients. Adding to these phenomena, anthropogenic forest clearing for agriculture, aquaculture, and tourism-based infrastructures leaves a minimal scope for enhancing blue carbon content in this region.

Analyzing the results and reviewing the existing literature, this study offers the following recommendations, which require decisive intervention from the government, non-government sectors, academics, and researchers:

- A comprehensive estimate of the total blue carbon stock of the Indian Sundarban through high spatial-resolution ground sampling is required. Eliminating the uncertainties by sampling throughout the spatial extent of the Indian Sundarban would enable us to derive the total carbon stock estimates and compute the social cost of carbon with higher confidence levels.
- To combat cyclone-induced damage to the mangrove floral stands, the state forest administration should strengthen its team to cater to the damaged trees by pruning them, covering exposed roots with soil, and clearing broken and malformed branches. Such care immediately after cyclonic storms would enhance the regeneration potential of the damaged trees.
- To alleviate the salinity stress in the Indian Sundarban, river rejuvenation in the upper reaches can always be a theoretical option that can solve many of these problems; however, in reality, it is challenging to execute, as these regions currently shelter thousands of inhabitants. Thus, planners and policy managers should explore avenues of channelizing freshwater into the central part of the Indian Sundarban through underground pipelines from the perennial flow of the Hooghly River.
- To combat the salinity-driven changes in the mangrove species assemblage, locating suitable areas and planting oligohaline species like *Heritera fomes*, *Nypa fruticans*, and *Sonneratia* sp. should be prioritized in the upcoming restoration programs. All mangrove restoration endeavors should focus on maintaining species diversity, as observed in the latest restoration effort after cyclone Amphan.
- Channeling freshwater from upper reaches through underground canals can alleviate nutrient and sediment deprivation. Arresting sea-level rise is beyond human control; however, illegal dredging activities should be controlled through strict law enforcement to combat sediment scarcity in this region.
- Overall, the local community has a considerable degree of awareness towards mangrove conservation as these mangroves protect them from natural hazards like tropical cyclones and storm surges. However, implementing payments against ecosystem services and carbon stock maintenance remains an alien concept in this region. The government should ensure that such initiatives see daylight and that the local stakeholders directly benefit from such programs, considering that conserving the Indian Sundarban can help India achieve 0.4% of its nationally determined contribution towards creating additional carbon sinks. Initiatives are also welcome to spread the significance of blue carbon sequestration among the commons, which can accentuate the need to conserve and protect this unique marine habitat.
- Law enforcement regarding the maintenance of estuarine water quality is virtually absent in this part of the world. This aspect needs immediate attention from the government to safeguard the mangroves and all biotic communities within this ecosystem. Integrating mangroves with aquaculture farming is another way out that needs the rigorous attention of the scientific community. Land conversion from mangroves to any other land-use class should be strictly prohibited, and the area coverage of the marine protected areas should be increased.

## Figures and Tables

**Table 1:** List of significant tropical cyclones that made landfall in the Sundarban region during the last two decades.

Name of the Cyclone	Landfall Date	Landfall Speed (km h^−1^)	Observations	References
Sidr	November 2007	215	1291 km^2^ forested area was severely affected.	[80]
			Tall plants like Sonneratia were most affected.	[88]
Rashmi	October 2008	85	258 km^2^ forested area was severely affected.	[80]
			The impact was much less than that of Sidr.	[89]
Aila	May 2009	110	53 km^2^ forested area was severely affected.	[80]
			The Indian Sundarban lost 21.6 km^2^ of forest cover.	[82]
Komen	July 2015	85	137 km^2^ forested area was severely affected.	[80]
Roanu	May 2016	85	152 km^2^ forested area was severely affected.	[80]
Bulbul	November 2019	137	The canopy density of almost 780 km^2^ area changed from very high (80–100%) to high (60–80%).	[91]
Amphan	May 2020	155	Dense mangrove cover shrank from 77% to 34%.	[92]
Yaas	May 2021	120	Widespread inundation in Sundarban.	[93]

**Table 2:** Significant observations by researchers regarding erosion-driven mangrove loss in the Indian Sundarban.

Duration of the Study	Observations	References
1973 to 2000	Indian and Bangladesh Sundarban combined witnessed an increase in 81.5 km^2^ of mangrove area between 1973 and 1990 and a loss of 152 km^2^ between 1990 and 2000, leading to a net loss of 70.5 km^2^ mangrove area between 1973 and 2000	[117]
1975 to 2013	A net mangrove area loss of 107 km^2^ occurred in the Indian Sundarban that led to a potential CO_2_ emission of 1567.98 ± 551.69 Gg	[118]
1984 to 2018	Indian and Bangladesh Sundarban combined witnessed a mangrove area loss of 136.77 km^2^ and an accretion of 62.17 km^2^, leading to a net loss of 74.6 km^2^	[119]
1986 to 2012	A net mangrove area loss of 124 km^2^ occurred in the Indian Sundarban due to erosion and submergence of southern sea-facing mangrove islands	[121]
2000 to 2017	A net mangrove area loss of 374 km^2^ occurred in the Indian Sundarban	[122]
2000 to 2020	110 km^2^ of mangrove loss due to erosion and 81 km^2^ gained through plantation and regeneration, leading to a net mangrove area loss of 29 km^2^ in the Indian Sundarban	[91]

## Data Availability

The data and observations discussed in this review are available in this paper and cited papers and articles.
